# Cardiac Embolism Following Abdominal Section

**Published:** 1895-08

**Authors:** C. C. Frederick

**Affiliations:** Buffalo, N. Y., Surgeon-in-Chief to the Buffalo Woman’s Hospital; Gynecologist to the Erie County Hospital.


### CARDIAC EMBOLISM FOLLOWING ABDOMINAL SECTION.

By C. C. FREDERICK, B. S., M. D., Buffalo, N. Y.,
Surgeon-in-Chief to the Buffalo Woman's Hospital; Gynecologist to the Erie County Hospital.

SOMEONE has said, "the unexpected always happens." Nowhere is the truth of this assertion more fully appreciated than among medical men, for there is always something new and unexpected turning up. As an illustration of one of the possible complications which may follow any operation, I wish to report one of death from cardiac embolism, following a section done for double tubo-ovarian abscesses.

Embolism of the heart, lungs or brain, during labor, after labor or accompanying phlebitis, is not rare. I have failed to find any reported cases of embolism following operations, the exciting cause being the operation itself. I think it must be rare.

The only other case of which I know occurred in June, 1891, in the clinic of Prof. Leopold, at Dresden, during the time that I was with him. She was a young woman under twenty-five years, whom he dilated slowly with laminaria tents for dysmenorrhea. The patient was strong and well nourished. Within twenty-four hours after the removal of the laminaria tent she suddenly developed dyspnea and cyanosis, which lasted for about six hours till she died. An autopsy was made the following day. A large clot was found obstructing a large branch of the pulmonary artery at a bifurcation. Prof. Leopold expressed his surprise that an embolus could originate from so slight a cause. He also stated that this was the first time he had seen embolism following an operation.

The history of my case is as follows :

Mrs. M., *æt.* 40, American, entered the Erie County Hospital May 30, 1894. She gave a history of pelvic inflammation several years before, from which she had suffered continuously and for which she sought relief. She was emaciated, had continuous pelvic pain, and had recurring attacks of severe paroxysms of pain and fever, which would keep her in bed for several days. She had the cachectic look which usually accompanies pus collections.

Examination of the pelvic contents showed large tender masses filling the entire pelvis and holding all the organs there firmly fixed. Abdominal section for removal of the diseased structures was recommended and the patient gave her consent to the operation. Her heart, lungs and kidneys were found to be healthy, but she was given tonics and strychnia hypodermically for several days in preparation for operation.

On June 13th, at 10.30 A. M., assisted by Dr. Hayd and the house staff, I removed the tubo-ovarian abscesses of each side, both of which were covered in and bound down by firm adhesions. Although the adhesions were thick and dense, the bowel was all separated without rupture. The meso-rectum, for about two inches of its course, just below the sigmoid, was torn through, but not to so great an extent that interfered with the nutrition of the gut. The only delay in the operation was caused by two small vessels deep in the pelvis, which bled freely till the patient was put into the Trendelenburg posture, when they were easily found and ligated.

The abdomen and pelvis were washed out with a warm normal salt solution, and a glass drainage-tube placed in Douglas's pouch. Drainage was used because one of the abscess sacs had ruptured during enucleation, flooding the abdomen with pus.

The patient rallied promptly and came out of the anesthetic without shock. In twenty hours the drainage-tube was removed. At about 5 P. M., June 14th, (thirty hours after operation,) she began to have dyspnea, a rapid and irregular pulse, with cyanosis. Auscultation over the heart showed a tumultuous action with confused murmurs, a veritable churning sound. Despite hypodermic stimulation with strychnia, digitalin and glonoin, she grew rapidly worse and died at 11.40 P. M. the same day, in six hours and forty minutes after the commencement of the attack.

The following day, June 15th, an autopsy was made by Dr. Francis Metcalfe. The pelvis and its contents were found free from any evidences of peritonitis. The bowels had adhered to the surfaces from which the diseased structures had been removed, completely sealing the rents in the peritoneal surfaces made necessary by the enucleation of the diseased appendages. The liver, spleen, kidneys and other abdominal viscera were normal. The pulmonary arteries contained no clots and the lungs were not diseased. The heart and its valves were normal. In the right ventricle was found a large clot which nearly filled the cavity, and which was entangled in the valves. The center of this clot was found to consist of a solid mass of fibrine, as large as a chestnut.

Evidently this clot caused the dyspnea and death. The fibrinous center of the clot probably had its origin in some of the pelvic veins, and had been carried into the ventricle and there became entangled in the valves.

64 RICHMOND AVENUE.

